# General practitioners’ perspectives on lifestyle interventions for cognitive preservation in dementia prevention

**DOI:** 10.1186/s12875-024-02566-3

**Published:** 2024-08-14

**Authors:** Josefine Kappe, Felix Wittmann, Melanie Luppa, Maria Isabel Cardona, Solveign Weise, Stephan Fuchs, Robert Philipp Kosilek, Linda Sanftenberg, Christian Brettschneider, Juliane Döhring, Catharina Escales, David Czock, Birgitt Wiese, Jochen René Thyrian, Wolfgang Hoffmann, Thomas Frese, Jochen Gensichen, Hans-Helmut König, Hanna Kaduszkiewicz, Steffi Gerlinde Riedel-Heller

**Affiliations:** 1 Institute of Social Medicine, Occupational Health and Public Health (ISAP), University of Leipzig, Phillip-Rosenthal-Str. 55, Leipzig, 04103 Germany; 2 Institute for Community Medicine, University Medicine Greifswald (UMG), Greifswald, Germany; 3 Institute of General Practice and Family Medicine, Martin-Luther-University Halle-Wittenberg, Halle, Saale, Germany; 4 Institute of General Practice and Family Medicine, University Hospital of LMU Munich, Munich, Germany; 5 Department of Health Economics and Health Service Research, University Medical Centre Hamburg-Eppendorf, Hamburg, Germany; 6 Institute of General Practice, University of Kiel, Kiel, Germany; 7 Department of Clinical Pharmacology and Pharmacoepidemiology, Heidelberg University Hospital, Heidelberg, 69120 Germany; 8 Institute for General Practice, Work Group Medical Statistics and IT-Infrastructure, Hannover Medical School, Hannover, Germany; 9 German Centre for Neurodegenerative Diseases (DZNE), site Rostock/ Greifswald, Greifswald, Germany; 10 Faculty V: School of Life Sciences, University of Siegen, Siegen, Germany

**Keywords:** Dementia, Alzheimer´s disease, Prevention, Risk factor, General practitioner

## Abstract

**Background:**

General practitioners (GPs) play a crucial role in identifying cognitive impairment and dementia and providing post-diagnostic care. This study investigates (1) how promising GP consider lifestyle changes to maintain cognitive performance in general, (2) GP beliefs about the power of modifiable health and lifestyle factors to maintain cognitive performance, and (3) whether those beliefs vary by GP age.

**Methods:**

As part of the AgeWell.de trial, GPs (*n* = 72) completed a process evaluation questionnaire assessing their perspectives on lifestyle changes to preserve cognitive performance in elderly patients. In greater detail, their perceived efficacy of established risk and protective factors was investigated using a 5-point Likert scale. Descriptive statistical analyses were performed for research question (1) and (2). Spearman´s rank correlations and ordinal logistic regressions were used to answer research question (3). All results were interpreted exploratively.

**Results:**

GPs rated the overall chance of lifestyle changes maintaining cognitive performance quite neutral with a median score of 3.0 (*IQR* = 2.0). They rated the efficacy of all the modifiable health and lifestyle factors high, with increase in physical and social activity ((*Mdn* = 5.0, *IQR* = 1.0) receiving the highest ratings with the narrowest range. Spearman's rank correlation indicated a significant positive relationship between age and the belief in “Optimization of nutrition” for preventing cognitive decline and dementia (*ρ* = .255, *p* = .041). However, ordinal logistic regressions showed no significant relationships between age and GP ratings of lifestyle change efficacy.

**Conclusion:**

These findings highlight the positive perception of GPs on the efficacy of modifiable health and lifestyle factors for preventing cognitive decline and dementia.

**Trial registration:**

The AgeWell.de trial is registered in the German Clinical Trials Register (DRKS; trial identifier: DRKS00013555, Registration Date 07 December 2017).

**Supplementary Information:**

The online version contains supplementary material available at 10.1186/s12875-024-02566-3.

## Introduction

Dementia stands as one of the most prevalent and severe mental disorders among individuals aged 60 and above, affecting approximately 1.8 million people in Germany alone by the end of 2021 [1]. Dementia is accompanied by a necessity for caregiving, institutionalisation, and ultimately premature mortality [2]. With projections indicating a doubling of the elderly population by 2050 [3], the public health concern associated with dementia is increasing, necessitating effective prevention strategies against the background of missing widespread pharmacological treatments [4].

The multifactorial nature of dementia reveals both unmodifiable factors like age and sex, as well as several modifiable risk and protective factors [5, 6]. The Lancet Commission on Dementia Prevention and Care has identified twelve modifiable factors contributing to approximately one-third of all dementia cases worldwide [6]. Targeting these twelve factors throughout life could presumably reduce the risk of dementia up to 40%, highlighting the potential for prevention [6]. These factors include low education, exposure to air pollution, hearing loss, traumatic brain injury, arterial hypertension, obesity, high alcohol consumption, diabetes mellitus, depression, physical inactivity, smoking, and social isolation.

Recognizing the urgency, the World Health Organization (WHO) emphasizes the priority of enhancing dementia literacy and proactive management of modifiable dementia risk factors, particularly in primary care, for maximizing the risk reduction potential [3, 7]. Serving as the first point of contact for health concerns, general practitioners (GPs) are essential in the initial identification of cognitive impairment and providing post-diagnostic care [8, 9]. In addition, the primary care system in Germany, in line with the Act to Strengthen Health Promotion and Prevention (Prevention Act – PrävG) [10], recognises GPs as promising health care providers for implementing interventions in real-world settings. It is expected that GPs take on a growing significance in the early detection, diagnosis, and ongoing care coordination for people with dementia [11]. Primary health services already offer opportunities to prevent non-communicable diseases [12]. Since the risk factors for dementia overlap with those of other non-communicable diseases [13], primary care professionals are well-equipped to participate in preventive measures for dementia as well. Despite their pivotal role, research indicates gaps in GP knowledge and confidence regarding dementia risk factors, hindering their ability to provide effective preventive guidance [11, 14]. Primary care research has focused on the role of GPs in diagnosing and managing dementia, rather than providing preventive advice [11].

Furthermore, it remains unclear whether sociodemographic characteristics, such as age, may influence GP perceptions. Older GPs, potentially with more work experience, may have different views on the effectiveness of dementia prevention compared to their younger counterparts. This could be due to their extensive clinical experience and exposure to a wide range of patient outcomes over time. Conversely, younger GPs might be more updated with recent research and contemporary medical practices, possibly influencing their openness to new preventive measures.

Therefore, this research aims to explore (1) how promising GP consider the lifestyle changes to maintain cognitive performance in general in older GP patients at increased risk for dementia, (2) GP beliefs about the power of established risk and protective factors to prevent cognitive decline and dementia and (3) whether those beliefs vary depending on the age of the GPs. Addressing these perceptions and understanding potential variations is essential for enabling GPs to offer effective preventive guidance and for developing targeted educational and support programs for GPs, contributing to the broader public health response to dementia.

## Methods

### Study design of AgeWell.de

This study analyses data from the AgeWell.de trial. AgeWell.de was a multicentre cluster randomized intervention trial designed as multi-component lifestyle intervention. Over a two-year intervention period, AgeWell.de aimed to target modifiable health and lifestyle factors associated with dementia in primary care patients with an increased risk of dementia. Participants, aged 60 to 77 years (*n* = 1030), were recruited by GPs at five study sites in Germany (Leipzig, Greifswald, Halle, Kiel and Munich). All GPs at these sites were eligible for participation, with a preference for those with established networks. No additional eligibility criteria were imposed on GP selection. GPs received advice on patient medication but were not further involved in the lifestyle intervention itself. Monetary incentives were provided to GPs for patient recruitment and data provision. The full study design rationale, recruitment procedure of the GPs and patients, and baseline characteristics have been described previously [15, 16].

### Process evaluation questionnaire

For the present analysis, we used data from the self-developed AgeWell.de process evaluation questionnaire (Appendix 1). This standardized questionnaire was designed to assess GP views on dementia risk reduction by lifestyle changes as comprised by the AgeWell.de-intervention, as well as the perceived effectiveness of various factors for preventing cognitive decline and dementia each on a 5-point Likert scale. Those factors considered are orientated to the modifiable risk and protective factors according to Livingston et al. [6]. Since the AgeWell.de-trial followed a pragmatic approach, aimed at feasible implementation in real world settings, GP views regarding the overall-chances for dementia risk reduction by lifestyle changes were also of great interest regarding future implementation of the intervention. The questionnaire was answered by the GPs at the end of the lifestyle intervention. These GPs were responsible for the recruitment of the participants in the AgeWell.de trial. As part of the follow-up examinations two years after baseline, the GPs received various documents by post (patients medication plans, questionnaires on diagnoses, etc.). The process evaluation questionnaire described in this study was sent at the same time. Due to dropouts of individual participants between baseline and follow-up examination (e.g. if practices had recruited only one patient), 6 GP practices dropped out between baseline and follow-up, leaving 117 GPs who received the questionnaire. The questionnaire was sent back to the respective study centre. For the present analysis, two main questions of the process evaluation were used. First, the GPs were asked how they assess the overall chance of lifestyle changes to maintain cognitive performance in older GP patients. GPs were asked to rate it on a 5-point Likert scale (range_low-high_ 1–5) with response option ranging from “very low” to “very high”. The analysis of this question is used to answer the research question (1) and (3). Second, they were asked how effective they perceived various factors for the prevention of cognitive decline and dementia to be. GPs were asked to rate them on a 5-point Likert scale (range_low-high_ 1–5) with response options ranging from “Does not apply at all” to “Applies completely”. The factors were optimization of nutrition; increase in physical activity; increase in social activity; cognitive training; education/lifelong learning; smoking cessation; weight reduction; reduction in alcohol consumption; intervention for depression; intervention for loss and grief; medication optimization regarding diabetes management, blood pressure control, reduction in anticholinergic medication; prevention of head injuries; reduction of air pollution and treatment of hearing loss. The analysis of this question is used to answer the research question (2) and (3).

### Trial registration

The AgeWell.de trial is registered in the German Clinical Trials Register (DRKS; trial identifier: DRKS00013555) and was carried out in accordance with the principles of the Declaration of Helsinki in its revised version from 2013.

### Statistical analysis

Since Shapiro–Wilk tests indicated that the variables are not normally distributed, descriptive statistical analyses were performed, calculating medians and interquartile ranges (IQRs) for the various modifiable health and lifestyle factors. These were used answering research question (1) and (2). To address research question (3) concerning the relationship between GP age and their beliefs about the effectiveness of risk and protective factors in preventing cognitive decline and dementia, a combination of Spearman rank correlation analyses and ordinal logistic regressions were conducted. The Likert-scale responses regarding GP beliefs were treated as ordinal variables due to their ranked nature. Spearman’s rank correlation was used to assess the relationship between the age of GPs and their beliefs about each specific risk and protective factor. This non-parametric method was chosen because it does not assume a normal distribution of the data, making it appropriate for ordinal data and the small sample size. For each item, Spearman’s rho (ρ) was calculated to measure the strength and direction of the association between age and the item response. To further analyse the influence of GP age on their beliefs, ordinal logistic regression analyses were performed for each item. Each item was treated as the dependent variable in separate ordinal logistic regression models, with age as the independent variable. The statistical significance level for all analyses was initially set at α = 0.05. All analyses were conducted using RStudio version 1.4.1717 [17] for Windows. All results were interpreted exploratively.

## Results

### Descriptive statistical analyses

**Fig. 1:**
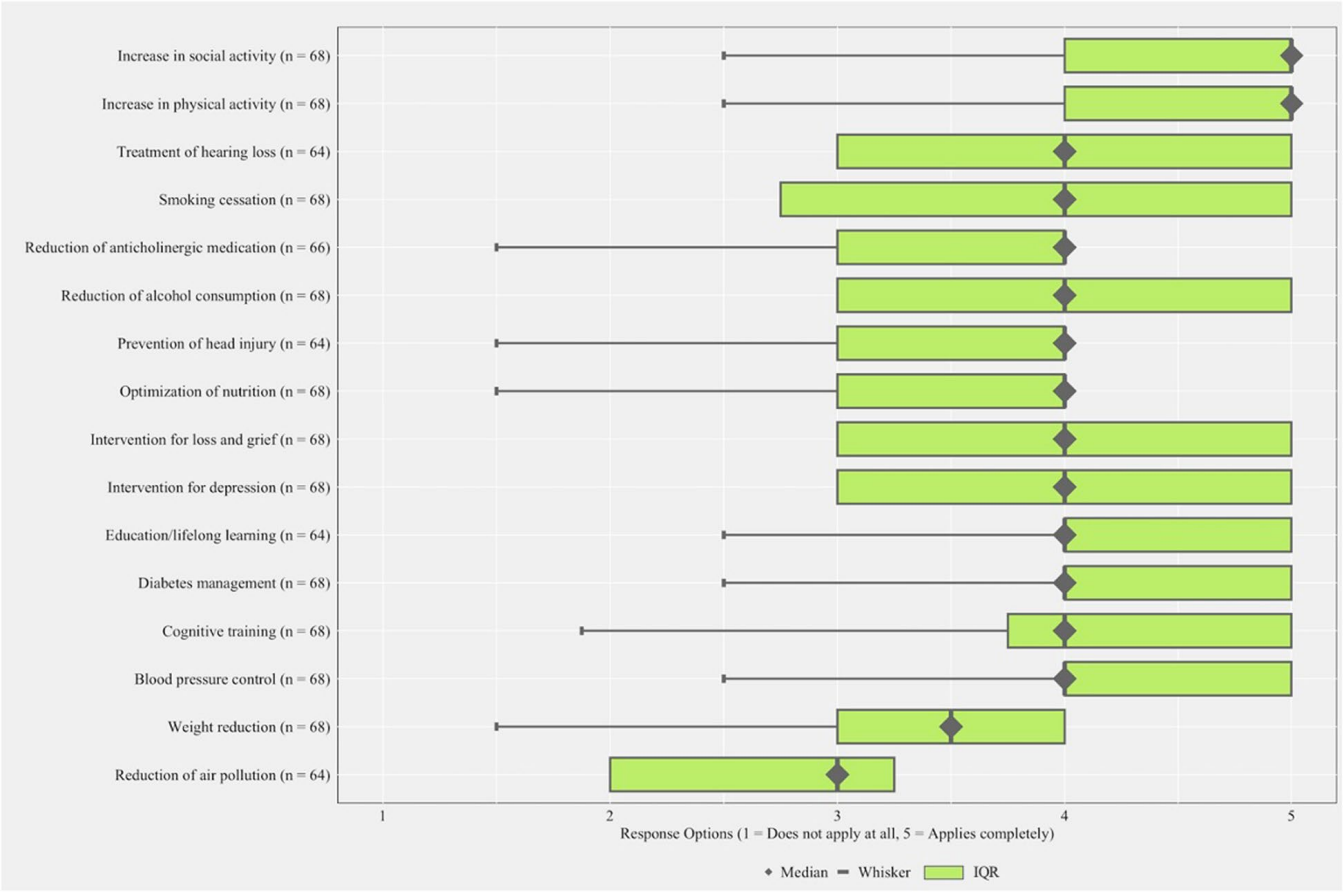
GPs perceptions on the promise of modifiable health and lifestyle factors for preventing cognitive decline and dementia (*n* = 72)

The final analytical sample consisted of *n* = 72 GPs (response rate 61.54%). 66 GPs provided information on their age with a mean age of 53.2 years. 67 GPs provided information on their gender. Of these, 52.24% (*n* = 35) were female. Figure 1 presents a Box-whisker plot of the distribution of the perception on the promise of established modifiable risk and protective factors to prevent cognitive decline and dementia by the GPs.

The median response for “overall chance for lifestyle change” was 3.0 (IQR = 2.0), indicating that the central tendency of responses was at the midpoint of the scale, with high variability. For both “Increase in physical activity” and “Increase in social activity “, the median was 5.0 (IQR = 1.0), reflecting a tendency towards higher agreement with the items and a narrow spread in responses. “Reduction of air pollution” had a median of 3.0 (IQR = 1.25), suggesting a central tendency at the midpoint of the scale with a narrow range of responses.

### Spearman´s rank correlation analyses

The correlation analyses between age of the GPs and their rating on the efficacy of modifiable health and lifestyle factors in prevention of cognitive decline revealed almost no statistically significant correlations. Only the variable “Optimization of nutrition” resulted in a moderate positive correlation (ρ = 0.255), which was statistically significant (*p* = 0.041). Detailed correlation results are shown in Table 1.

**Table 1:** Relationship between GP age and their rating on the efficacy of modifiable health and lifestyle factors in prevention of cognitive decline dementia (*n* = 72)

Variable	Ordinal logistic regression				Spearman´s rank correlation	
	*β*	SE (β)	*t*	*p*	ρ	*p (*ρ)
Overall chance for lifestyle change (*n* = 66)	-0.038	0.026	-1.468	.142	-0.167	.192
Increase in physical activity (*n* = 68)	0.007	0.025	0.294	.768	0.071	.577
Increase in social activity (*n* = 68)	0.010	0.025	0.401	.689	0.089	.482
Education/lifelong learning (*n* = 64)	0.005	0.025	0.202	.834	0.045	.728
Cognitive training (*n* = 68)	-0.007	0.024	-0.306	.759	-0.020	.872
Blood pressure control (*n* = 68)	0.046	0.026	1.746	.081	0.240	.054
Diabetes management (*n* = 68)	0.042	0.026	1.583	.113	0.222	.076
Intervention for depression (*n* = 68)	-0.036	0.025	-1.424	.154	-0.163	.195
Intervention for loss and grief (*n* = 68)	-0.015	0.024	-0.629	.529	-0.084	.508
Reduction of anticholinergic medication (*n* = 66)	0.010	0.025	0.406	.685	0.074	.566
Treatment of hearing loss (*n* = 64)	-0.007	0.024	-0.296	.767	-0.063	.629
Reduction of alcohol consumption (*n* = 68)	0.026	0.025	1.030	.303	0.156	.216
Prevention of head injury (*n* = 64)	-0.035	0.025	-1.404	.160	-0.180	.165
Smoking cessation (*n* = 68)	0.041	0.025	1.654	.098	0.234	.061
Optimization of nutrition (*n* = 68)	0.047	0.025	1.825	.068	0.255	.041*
Weight reduction (*n* = 68)	0.044	0.025	1.758	.079	0.243	.051
Reduction of air pollution (*n* = 64)	-0.008	0.024	-0.350	.726	-0.048	.716

The GPs provided ratings on a 5-point Likert scale, for the overall chance for lifestyle change ranging from “very low” to “very high”, scores 1–5 with higher scores indicating a higher chance. For the efficacy of modifiable health and lifestyle factors ranging from *“Does not apply at all”* to *“Applies completely”,* scores 1–5 with higher scores indicating a higher perceived efficacy

*β* regression coefficient for age*, SE* (β) Standard error for β, *ρ* Spearman´s rho

^*^indicates *p* < .05

### Ordinal logistic regression

The ordinal logistic regression analyses neither reveal a significant relationship between age and how GPs consider the lifestyle changes to maintain cognitive performance in general nor between age and each modifiable health and lifestyle factor.

## Discussion

### Main findings

This study primarily aimed to assess how GPs perceived the overall chance of lifestyle changes to maintain cognitive performance, finding that GPs were neutral on this matter. Secondly, this study aimed to investigate GPs perceptions on the promise of modifiable health and lifestyle factors for preventing cognitive decline and dementia. GPs were moderately to highly optimistic in the positive effect of individual risk and protective factors, with increase in physical activity and increase in social activity receiving the highest ratings. It is not possible to discuss the exact order of effectiveness assumptions of the GPs because the differences between factors are small. Ordinal logistic regression analyses were conducted to examine whether GP age was associated with their beliefs about the effectiveness of various risk and protective factors in preventing cognitive decline and dementia. Overall, none of the ordinal logistic regressions yielded significant results, indicating that age did not have a statistically significant effect on GP beliefs about the various factors studied. This suggests that the age of GPs may not be a decisive factor influencing their engagement with modifiable health and lifestyle factors in preventing cognitive decline. The Spearman’s rank correlation analyses indicated that age was only significantly associated with one of the rating items, specifically the “Optimization of nutrition.” This variable showed a moderate positive correlation with GP age (*ρ* = 0.255, *p* = 0.041), suggesting that older GPs were more likely to rate the efficacy of nutrition optimization high. This finding contrasts with the results from the ordinal logistic regression analyses. The discrepancy between the correlation and regression results can be attributed to several factors. The regression model, which included age as the only predictor, may have been too simplistic to capture more nuanced relationships. The lack of significant results in the regression analysis could suggest that age alone does not sufficiently explain variations in beliefs about the efficacy of different factors. In contrast, the Spearman correlation examines the direct, unadjusted association between age and each belief, potentially revealing relationships not evident when controlling for other factors. It is important to acknowledge that given the number of analyses conducted, this finding may be a false positive or due to chance, as the significance level was set at 0.05.

### Comparison with existing literature

The study’s primary focus aligns with the broader discourse on proactive healthcare. The revelation of the increase of physical activity was identified as one of the most promising intervention by GPs, which echoes in existing literature [6]. Numerous studies have consistently demonstrated a strong association between physical activity and a reduced risk of developing dementia. This evidence comes from both observational studies [18, 19], which show that individuals who engage in regular physical activity have lower rates of dementia, and intervention studies [20–22], which have shown that exercise programs can improve cognitive function and reduce the risk of cognitive decline. Unlike some other preventive measures for dementia, such as certain medications or specialized interventions, physical activity is accessible to almost everyone regardless of age, socioeconomic status, or location. It does not necessarily require expensive equipment or extensive resources, making it a highly feasible and cost-effective preventive strategy. Encouraging patients to increase their physical activity empowers them to take an active role in maintaining their own brain health and reducing their risk of dementia. It promotes a sense of personal responsibility for one’s health and well-being, which can lead to sustained behaviour change and long-term benefits. Overall, the wealth of scientific evidence supporting the benefits of physical activity, its accessibility and affordability, its potential to empower individuals in their own health management, and its integration into daily life make it a highly promising factor for preventing dementia, as recognized by GPs [23–25]. Comparing our results with Cations et al. [26], who indicated limited public awareness of the potential for dementia prevention and treatment, this study indicates that GPs possess a high level of knowledge regarding the efficacy of various modifiable factors, reflecting their awareness of multifaceted approaches to cognitive decline prevention.

### Strengths and limitations

To date, there is little evidence about how promising GPs rate established risk and protective factors for cognitive decline and dementia. Therefore, this study offers valuable insights into how effective the GPs consider these factors to be. However, this study has several limitations. Potential selection bias and a lower response rate may impact the findings’ generalizability, as the GPs participating in the AgeWell.de study could be those who exhibit a higher level of commitment to dementia care. Additionally, is has to be mentioned that the questionnaire was administered at the end of the trial. Although the GPs were not involved in the intervention itself, nor did they know through blinding procedure whether their patients were in the intervention or control group, it can be assumed that the GPs may have gained a certain familiarity with the topic through conversations with the participants about the content of the study. Although the reasons for non-participation were not investigated, factors such as time constraints or lack of interest may have influenced GP participation. Additionally, there were no distractors, i.e. factors that are not proven risk factors at all, to check whether the GPs really know what they are answering and do not assume from the outset that everything is a risk factor and that they just want to impress with their answers. This research considered the responses on the 5-point Likert scales as continuous variables, a common practice when these scales are perceived to represent ordered categories along a continuum and when the scale is finely graded enough to warrant such treatment [27]. While Likert scales are technically ordinal, treating them as continuous variables offers several advantages including the ability to calculate summary statistics and conduct statistical analyses such as correlation and regression [28]. Nonetheless, it is crucial to acknowledge the limitations of this method, including assumptions of uniform spacing between scale points, potential loss of information from collapsing ordinal categories into a continuous scale, and the risk of biased estimates if underlying assumptions are violated [29]. Moreover, it is important to consider that the responses clustered around the middle of the Likert scale, which is a known tendency with such scales [29]. This clustering suggests that GPs might have similar positive estimates of efficacy for most interventions, especially those they can directly influence. The lower rating for air pollution control, a factor beyond their direct control, underscores this point. This may reflect a general tendency among physicians to align with commonly accepted views, such as the belief that a healthy lifestyle benefits brain health. Future research should address whether this clustering is due to genuine consensus or a reflection of social desirability bias.

### Implications for research

As this study only investigates whether perceptions vary depending on GP age, future research could include additional factors, such as years of experience, practice location, and practice type. Longitudinal studies tracking changes in GP perceptions over time and examining how these perceptions influence their clinical practice could provide insights into the stability of GP attitudes towards dementia prevention strategies.

Besides the current quantitative approach, future research could benefit from integrating qualitative methods, such as interviews or focus groups, to investigate the underlying reasons for GP perceptions. This might provide valuable insights to inform targeted interventions aimed at enhancing their engagement in prevention efforts. Qualitative research could uncover underlying reasons for GP beliefs, identify potential barriers to implementing preventive measures, and explore the contextual factors influencing their perceptions. This approach would complement our findings and provide a deeper understanding of the complexities involved in primary care dementia prevention strategies.

Interestingly, while most of the component interventions were rated relatively high, the overall chance for lifestyle change was rated lower at *Mdn* = 3.0 (IQR = 2.0). This suggests that although GPs recognize the potential of individual lifestyle factors to prevent cognitive decline, they may be skeptical about the feasibility of achieving significant lifestyle changes in practice. Given that a multifactorial approach is likely to be most effective in reducing the risk of dementia, this discrepancy highlights the need for strategies to enhance the implementation of combined lifestyle interventions and design them more targeted on specific risk profiles. Moreover, considering the differential importance of risk factors at various points in the life course as highlighted by the Lancet Commission, it would be beneficial to understand GP views on different potential interventions across different life stages. This insight could reveal how willing GPs are to address these issues with their patients at various ages. Since the AgeWell.de trial included patients aged 60–77, it is crucial to clarify whether GP views were specifically sought for this age group or for earlier ages as well. Future research should aim to capture GP perspectives on interventions for different age groups, thereby informing more age-specific dementia prevention strategies. Addressing these implications can advance dementia prevention strategies in primary care, ultimately improving the brain health and quality of life of elderly patients.

## Conclusion

This research sheds light on the perceptions of GPs regarding the overall chance of lifestyle changes and modifiable health and lifestyle factors in preventing cognitive decline and dementia. GPs generally hold a positive view on the effectiveness of these interventions, particularly prioritizing physical and social activities, whereas age did not significantly impact these perceptions overall. These findings underscore the pivotal role of GPs in dementia prevention and highlight the need for continued exploration and support of their proactive healthcare practices.

### Supplementary Information

Supplementary Material 1

## Data Availability

The datasets cannot be accessed publicly due to privacy constraints. However, researchers can obtain individual participant data, once de-identified, by submitting a well-structured proposal to the AgeWell.de steering committee (contact: steffi.riedel-heller@medizin.uni-leipzig.de). Upon approval of the proposal, access to the datasets can be facilitated. Requests for dataset access should be directed to SR-H, steffi.riedel-heller@medizin.uni-leipzig.de.

## Abbreviations

GP: General practitioner
WHO: World Health Organization
