# Supplementary material for: General practitioners’ perspectives on lifestyle interventions for cognitive preservation in dementia prevention
Source: BMC Prim Care. 2024 Aug 14;25:301. doi: 10.1186/s12875-024-02566-3 (PMC11323454; doi:10.1186/s12875-024-02566-3)
Supplement: Supplementary file 1 — Supplementary Material 1 [file 12875_2024_2566_MOESM1_ESM.pdf]

AgeWell.de – Process Evaluation for Participating Primary Care Physicians

Age of the primary care physician (in years): ____________ **Gender:** ☐male ☐ female

Part 1: Effort

**Please estimate the total effort as a participating primary care physician taking part in the study AgeWell.de.**				
**very low**	**low**	**neutral**	**high**	**very high**
☐	☐	☐	☐	☐

**Please estimate the following aspects of your effort in detail:**					
	**very low**	**low**	**neutral**	**high**	**very high**
effort for screening (selection criteria, CAIDE-score)	☐	☐	☐	☐	☐
effort for explaining/getting agreement	☐	☐	☐	☐	☐
effort in the process of getting questions from the patient	☐	☐	☐	☐	☐
effort in the process of feedback for the studies team	☐	☐	☐	☐	☐

Part 2: Chances

How do you assess the chances of making lifestyle changes to maintain cognitive performance for older general practitioner patients?				
**very low**	**low**	**neutral**	**high**	**very high**
☐	☐	☐	☐	☐

How promising do you think the following aspects are for preventing cognitive decline and dementia?					
	**no effect**	**almost no effect**	**some effect**	**has an effect**	**has a strong effect**
Optimizing nutrition	☐	☐	☐	☐	☐
Increasing physical activity	☐	☐	☐	☐	☐
Increasing social activity	☐	☐	☐	☐	☐
cognitive training	☐	☐	☐	☐	☐
education / life long learning	☐	☐	☐	☐	☐
Stopping smoking	☐	☐	☐	☐	☐
Losing weight	☐	☐	☐	☐	☐
Reducing alcohol consumption	☐	☐	☐	☐	☐
Intervention in depression	☐	☐	☐	☐	☐
Intervention in loss and grief	☐	☐	☐	☐	☐
**Optimizing medication:**					
Diabetes control	☐	☐	☐	☐	☐
Blood pressure control	☐	☐	☐	☐	☐
Reduktion of anticholinergic medication	☐	☐	☐	☐	☐
Prevention of traumatic brain injuries	☐	☐	☐	☐	☐
Reducing air pollution	☐	☐	☐	☐	☐
Treating loss of hearing	☐	☐	☐	☐	☐
